# Supplementary material for: A randomized clinical trial of a new anti–cervical stenosis device after conization by loop electrosurgical excision
Source: PLoS One. 2021 Jan 20;16(1):e0242067. doi: 10.1371/journal.pone.0242067 (PMC7816995; doi:10.1371/journal.pone.0242067)
Supplement: S1 File — (DOC) [file pone.0242067.s002.doc]

**
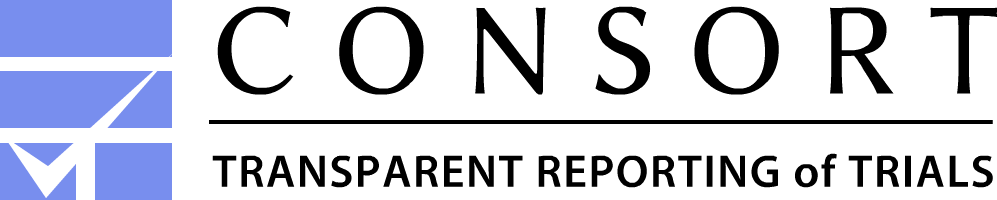
**

**CONSORT 2010 Flow Diagram – Phase III**

**Allocation**

**Analysis**

**Follow-Up**

**Enrollment**

Assessed for eligibility (n=260)

Excluded (n=20)

♦  Not meeting inclusion criteria (n=18)

♦  Declined to participate (n=2)

♦  Other reasons (n=0)

Analysed

- 1^st^ follow up (n=111)
- 2^nd^ follow up (n=102)
- 3^rd^ follow up (n=99)
- 4^th^ follow up (n=97)

♦ Excluded from analysis (n=23)

- (lost to follow up) (n=16)
- (cancer) (n=7)

Lost to follow-up (n=16)

Discontinued intervention (Cancer) (n=7)

DUDA group

Allocated to intervention (n=120)

♦ Received allocated intervention (n=120)

Lost to follow-up (n=16)

Discontinued intervention (Cancer) (n=9)

NO DUDA group

Allocated to intervention (n=120)

♦ Received allocated intervention (n=120)

Analysed

- 1^st^ follow up (n=110)
- 2^nd^ follow up (n=100)
- 3^rd^ follow up (n=100)
- 4^th^ follow up (n=95)

♦ Excluded from analysis (n=25)

- (lost to follow up) (n=16)
- (cancer) (n=9)

Randomized (n=240)
