# Supplementary material for: A randomized clinical trial of a new anti–cervical stenosis device after conization by loop electrosurgical excision
Source: PLoS One. 2021 Jan 20;16(1):e0242067. doi: 10.1371/journal.pone.0242067 (PMC7816995; doi:10.1371/journal.pone.0242067)
Supplement: S2 File — (DOCX) [file pone.0242067.s003.docx]

**Randomized Clinical Trial of a new Anti–Cervical Stenosis Device after Conization by Loop Electrosurgical Excision**

Department of Gynecologic Oncology and Prevention at Barretos Cancer Hospital

**Principal Investigator**

Marcelo de Andrade Vieira

**Researches**

Ricardo dos Reis

Audrey Tieko Tsunoda

Carlos Eduardo da Cunha Mattos Andrade

Talita Garcia do Nascimento

José Humberto Tavares Guerreiro Fregnani

The study was registered at clinicaltrials.gov with ID Number NCT02500966.

**URL:** <https://clinicaltrials.gov/ct2/show/NCT02500966?term=DUDA&draw=2&rank=1>

**Conflicts of Interests and Sources of Funding:**

The authors have declared no conflicts of interests.

Barretos, March 2015 (version 1.3)

Index

1- Introduction 4

2- Justification 6

3- Objectives

3.1 Primers 6

3.2 Secondaries 6

4 Materials and Methods 7

4.1 Study design 7

4.2 Study location 7

4.3 Inclusion Criteria 7

4.4 Exclusion Criteria 7

4.5 Description of device 9

4.6 Description of the high-frequency surgery (CAF) conization technique 11

4.7 Procedure for placing the endocervical device (DUDA) 12

4.8 Criteria for not placing or removing the device 13

4.9 Study steps 13

4.9.1 First stage: security analysis 14

4.9.2 Second stage: analysis of effectiveness 14

4.10 Study outcomes 15

4.10.1 Device security 15

4.10.2 Effectiveness 17

4.10.3 Quality of life 18

4.11 Study interruption rules 18

4.12 Study visits and data collection 19

4.12.1 Initial visit (baseline) 19

4.12.2 Operative and postoperative visits 19

4.12.3 Follow-up visits 20

4.12.4 Surgery information 20

5 Sample Calculation 21

5.1 First stage 21

5.2 Second stage 22

6 Statistical Analysis 22

7 Independent data monitoring committee 23

8 Ethical Aspects 23

8.1 Special thematic area 24

8.2 Consent Form 24

8.3 The weighting the risks and benefits 24

8.4 Biological material bank 24

8.5 Infrastructure and human resources 25

8.6 Data confidentiality 25

8.7 Guarantee of access to the search result 25

9 Detailed Budget 25

10 Schedule 26

11 Bibliography 27

1. INTRODUCTION

The term high-grade cervical intraepithelial neoplasia (CIN) is used to define lesions previously referred to as moderate dysplasia (CIN II) and severe dysplasia / in-situ carcinoma (CIN III) (1).

Literature reviews regarding the natural history of these lesions indicate that 43% of untreated NIC II lesions will regress in the absence of treatment, while 35% will persist and 22% will progress to in-situ carcinoma or invasive carcinoma (2). In comparison, 32% of NIC III lesions will regress spontaneously, 56% will persist and 14% will progress (1, 3).

The treatment of high-grade CIN (CIN II, III) is divided according to the patient's colposcopic evaluation. In cases of satisfactory colposcopy (visible squamous-columnar junction), both ablative and excisional methods are adequate (4). Ablative methods include cryotherapy, laser vaporization, electrocautery, diathermy, cold coagulation, while excisional methods include LEEP (excisional electrosurgical loop) also called High-Frequency Surgery (CAF), laser conization and cold conization.

It is important to emphasize that excision procedures allow a detailed assessment of all excised tissue, thereby reducing the risk that a micro-invasive lesion or occult invasive carcinoma is inadvertently treated as a pre-invasive lesion. When the colposcopic evaluation is unsatisfactory (squamous-columnar junction not visible) there is a chance of up to 7% of the diagnosis of occult invasive carcinoma in the conization specimen by a biopsy showing NIC II/III. Thus, diagnostic conization procedures that allow pathological examination of the tissue of the endocervical canal are usually used for women with biopsies confirming NIC II/III who have an unsatisfactory colposcopic evaluation.

The complications inherent to conization involve stenosis of the cervical canal, amenorrhea, dysmenorrhea and deep dyspareunia. (5)

1.1. Cervical canal stenosis

Stenosis of the cervical canal is the most important complication due to the clinical repercussions that can vary from menstrual cramps followed by hematometrium, infertility and the impossibility of early detection of recurrence and/or recurrence of the premalignant lesion (5, 6).

Studies show a rate of cervical canal stenosis ranging from 3 to 25% after laser conization and 1.3 to 19% when performed with LEEP (5-10). It can be diagnosed within 28 months of conization. (6)

Studies indicate that the variables of wide excision of the entire endocervix (RR 5.07) and extension of the excision of> 2cm (RR 1.95) are associated with a high risk of cervical stenosis after conization. (6)

Although many studies report cervical stenosis after conization, few studies in the literature describe techniques for treating endocervical canal stenosis. When describing some type of intervention, the study is not randomized and with a small number of patients. Among the techniques described there are reports of the use of devices that maintains the patency of the cervical for a brief period in order to avoid restenosis of the canal and maintain the patient's fertility or follow-up in women of non-fertile ages. (11, 12) (13)

Analysis of studies that used some type of device to prevent cervical stenosis

| Authors | Year | N | Device | Length of stay device | Type of study | Post device stenosis rate | Follow-up /  Complications  (N) |
| --- | --- | --- | --- | --- | --- | --- | --- |
| Nasu k et al | 2010 | 4 | DIU-Nylon | 3 months | Case report | 0 | Does not describe/ 0 (zero) |
| Luesley, DM et al | 1990 | 33 | stent | 2 weeks | Pilot Study | 6% | 6 months /  0 (zero) |
| Dorothee Grund et al | 2007 | 1 | Stent Vascular | 9 months | Case report | 0% | 11 months/  0 (zero) |

Table 1

As there is no indication and consensus procedure in the literature to prevent stenosis of the endocervical canal, this study proposes and uses a new device created at the Barretos Cancer Hospital (HCB), called the Uterine Endocervical Canal Dilatation Device (DUDA) (figure 1) in patients undergoing conization in an attempt to assess and avoid the post-CAF stenosis index.

2. JUSTIFICATION

Currently, there is no standard and consensual standard procedure for the prevention and treatment of cervical canal stenosis after a conization procedure. There is a limited number of clinical trials, all non-randomized, that use temporary devices in an attempt to avoid this type of complication (11, 12). Although the conclusions of these studies are favorable, more robust evidence about the safety and efficacy of these methods is still lacking. It is necessary to explore with greater scientific rigor the application of devices in the cervical canal to prevent its stenosis.

3. OBJECTIVES

3.1. Primary objective:

To evaluate the effectiveness of a new cervical device called DUDA in preventing late cervical canal stenosis in women undergoing conization with a high-frequency scalpel.

3.2. Secondary objectives:

3.2.1. Assess the safety of using the device;

3.2.2. Compare the quality of life in women with and without the device after surgery;

3.2.3. Compare the rate of visualization of the squamous-columnar junction (JEC) in women who used the device or not (3 and 6 months after surgery);

3.2.4. Describe menstrual periods in women who used device placement after surgery.

4. MATERIALS AND METHODS

4.1. Study design:

This is a randomized, open, parallel-group clinical trial to investigate the safety and efficacy of a new cervical device called DUDA (Uterine Device for Dilatation of the Endocervical Canal). As this is an intervention in which the doctor must know the result of the randomization for implantation of the device, he will not be blind. The study will be carried out sequentially in two stages, the first being intended to exclusively evaluate the safety of using the device and the second to evaluate safety and effectiveness.

4.2. Study Location:

The study will be carried out in the Oncology and Prevention Gynecology Departments of the Barretos Cancer Hospital/Pio XII Foundation (Barretos, SP).

4.3. Inclusion criteria:

For inclusion, all criteria must be present:

a) Histopathological diagnosis of NIC2/3 on cervical biopsy;

b) Indication for performing CAF (high-frequency surgery);

c) Age between 18 and 65 years;

4.4. Exclusion criteria:

For exclusion, any of the following criteria must be present:

a) Pregnant women before CAF;

b) Previous conization / CAF history;

c) Women who do not understand or accept the study;

d) Women subjected to a regime that determines significant vulnerability to the participant (ex: prisoners, indigenous, etc.)

4.5. Description of the Endocervical Canal Dilatation Device (DUDA):

It is a plastic device (polyacetal) designed by the responsible researcher, made by hand on a lathe through the wear of a cylindrical part.

Polyacetal is a plastic material. This material is unique, pure (without additives) with exceptional dimensional stability, low friction coefficient, low moisture absorption and high abrasion resistance. It maintains characteristics when immersed in hot water. High durability, is resistant to biological attacks and impacts, and can even hold weights indefinitely. It also has low weight, high melting point, good impact resistance, high lubricant, thermal insulation, low friction coefficient, high resistance to wear and chemical agents, thus being able to substitute bronze, brass, aluminum with advantages. In addition to the advantages presented, this material is inexpensive. It does not have a porous surface and, therefore, the concentration of substances is inert. As a consequence, it does not generate biofilm, which reduces the risk of infections associated with it. Before use, the device will be sterilized according to the Institution's operational procedure.

Cylindrical at one of its ends, with 4 holes (where the points will be anchored), 2.5 cm in diameter, accompanied by a cylindrical rod with a diameter of 4 mm, central lumen and 2 cm in length (Figures 3, 4 and 5). The nail is introduced into the endocervical canal and the larger cylindrical part is in contact with the external surface of the cervix.


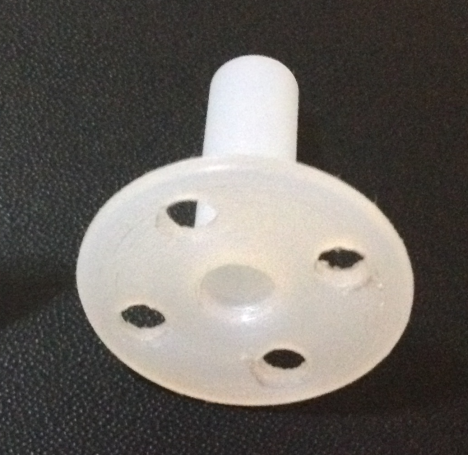


Figure 3: Anterior view of the device (DUDA).


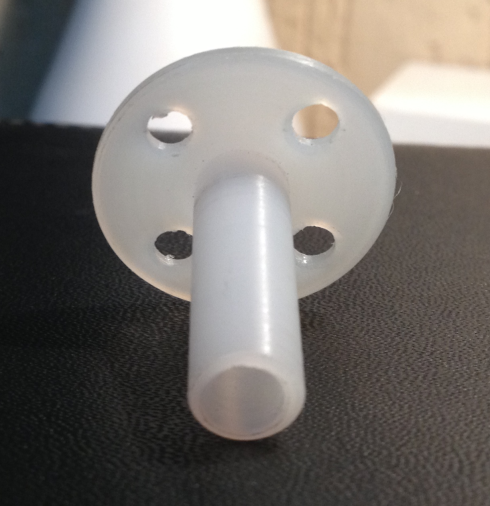


Figure 4: Rear view of the device (DUDA).


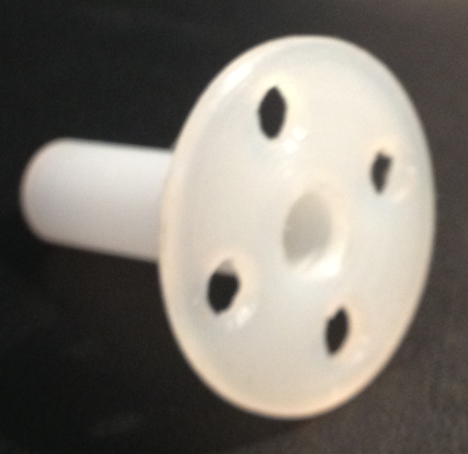


Figure 5: Side view of the device (DUDA).

4.6. Description of the conization technique with high-frequency surgery (CAF):

           High-frequency surgery (CAF) is performed by means of a diathermic loop (Figures 1 and 2) connected to an energy source, in order to remove all the so-called transformation zone of the cervix. The size of the resection can be programmed by the surgeon based on the size of the colposcopic lesion and from there choose the best loop size to be used. It is worth remembering that this procedure is part of the conventional treatment of cervical intraepithelial neoplasms and its indication regardless of the acceptance or not of the research participant. Therefore, CAF does not represent the experimental part of the study.

The CAF lasts approximately 20 minutes and follows the technical steps described below:

1) Intravenous sedation;

2) Positioning the patient in the supine position on Allen's leggings and in the lithotomy position;

3) Asepsis with degerming iodine throughout the perineal and vaginal region. If the patient has an allergy to iodine, asepsis is performed with chlorhexidine gluconate solutions;

4) Placement of sterile surgical drapes;

5) Introduction of the vaginal speculum and identification of the cervix;

6) Passage of acetic acid and Lugol´s iodine in the cervix and vaginal walls to identify positive acetowhite and iodine areas;

7) Local anesthesia with 2% lidocaine with a vasoconstrictor, infusing 2 ml in each quadrant of the cervix (3, 6, 9 and 12h);

8) Passage of the diathermic loop connected to the generating source in “cut” mode (level 60) in a single pass, with or without enlarging the endocervical canal (depending on the indication of each case);

9) Hemostasis with a "ball" electrode connected to the generating source in "coagulation" mode (level 60).

After this surgical procedure, the patient remains in the hospital for a few more hours for recovery from anesthesia and observation of eventual postoperative bleeding. The patient is discharged from the hospital with guidance on the use of analgesics (if necessary) and to abstain from sexual intercourse for at least 30 days due to the risk of bleeding during intercourse.

4.7 Procedure for placing the endocervical device (DUDA):

After the end of the CAF, and with the patient still positioned on the operating table, the surgeon will place the DUDA device on the cervix. Device deployment is quick and requires no more than 5 minutes. It should be noted that this procedure represents the experimental part of the study.

The surgeon will carefully insert the nail into the endocervical canal until the flat circular surface of the device rests on the cervix. By means of the simple suture in four points (2-0 polypropylene thread, atraumatic needle), the device will be fixed to the surface of the cervix. In the event that the residual neck has a very flat surface, the device may be attached to the lateral fornices.

4.8 Criteria for not placing or removing the device:

Even if the participant has signed the Free and Informed Consent Form and the randomization has already been carried out, the DUDA device will not be placed on patients who have any complications during the performance of the CAF and that require additional procedures, such as bed bleeding. surgical procedure requiring hemostatic procedures with suture and/or use of a vaginal tampon.

Participants whose anatomopathological report of the conization surgical specimen demonstrates invasive cervical carcinoma will have the device removed before the deadline and will be referred for appropriate cancer treatment.

The early removal of the device may be carried out if, after its placement, the researcher identifies potential risk to the research participant or also at the request of the same.

4.9 Study steps:

This is a study whose main objective is to evaluate the safety and effectiveness of a new therapeutic device. The study design foresees the realization of two distinct sequential steps. The first stage aims to determine the safety of using the DUDA device in initially 25 women. Concluding that the device is safe for the participants, we will proceed to the second stage, in which, in addition to safety, the effectiveness of the device in preventing post-conization endocervical canal stenosis (CAF) will be evaluated in a study randomized, open and parallel study with 240 women (120 in each arm). This study design aims to bring greater security to the research participants, avoiding recruiting a large number of women unnecessarily.

4.9.1. First step: security analysis

In this stage of the study, 25 women will initially be included to determine the safety of the device during its use and up to 30 days after its removal. The main safety events to be considered in this analysis will be vaginal hemorrhage and uterine infection, both recorded according to the CTCAE version 4.0 criteria. Other adverse events that the researcher judges to be related to the use of the device will also be recorded (causal link at least possible).

The device will be considered unsafe and, consequently, the study will be interrupted if there is a single adverse event grade 3 or higher.

If no grade 3, 4 or 5 adverse events are recorded in this series of 25 cases, the study will proceed to the second stage after evaluation and recommendation by the Independent Data Monitoring Committee and also after analysis and approval of the partial (safety) report by the CEP/CONEP system.

4.9.2. Second step: analysis of the effectiveness

The participants in the first stage will not be part of the second. Although the main objective of this step is to evaluate the effectiveness of the device in preventing post-conization cervical canal stenosis, safety and quality of life issues will also be analyzed. The observation period for the second stage will be two years.

In the second stage, 240 participants will be recruited randomly balanced 1: 1 in two groups: one who will receive the DUDA device after CAF and another who will not receive it. Randomization, active search and patient management will take place at the outpatient clinic after the signature of the TLCE by the nurse responsible for clinical research in the department and will be carried out in blocks of 4, through the REDCap program.

4.10. Study outcomes:

4.10.1. Device security:

The safety analysis will be performed up to 30 days after surgery. The definitions of CTCAE (Common Terminology Criteria for Adverse Events) version 4.0 will be adopted as a reference for the security assessment of the device. For that, two main safety events will be considered during its use: vaginal bleeding and uterine infection. Below are the gradations for the two main security events defined by CTCAE version 4.0.

a) Vaginal bleeding:

- Grade 1: mild bleeding, identified on clinical examination or imaging study. No intervention is indicated;
- Grade 2: moderate bleeding; Medical intervention is indicated;
- Grade3: bleeds, intense; need for a blood transfusion; radiological or endoscopic intervention is indicated;
- Grade 4: bleeding determines life-threatening; urgent surgical intervention is indicated;
- Grade 5: death from bleeding.

b) Uterine infection:

- Grade 1: (there is no category for this event);
- Grade 2: moderate symptoms; oral intervention is indicated (eg: antibiotics, antifungal, antiviral);
- Grade 3: intravenous intervention is indicated (eg: antibiotics, antifungal, antiviral); radiological intervention or surgical intervention are indicated;
- Grade 4: infection determines life-threatening; urgent surgical intervention is indicated;
- Grade 5: death from bleeding.

Other events in which the researcher believes that there is a possible, probable or definitive relationship with the device (during or after its use) other than the two mentioned above will also be classified according to CTCAE version 4.0. Information about all adverse events provided voluntarily by the research participant, discovered by the researcher's questions or detected by physical examination, laboratory tests or other means, will be collected, recorded in the medical record and monitored as appropriate.

Clinical conditions/illnesses present before starting study treatment are considered adverse events when they worsen after the start of study treatment (any procedure specified in the protocol). Adverse events that occur before the study treatment begins, but after signing the informed consent form will be recorded in the medical record. Laboratory values ​​or abnormal test results are only adverse events if they induce clinical signs or symptoms or require therapy, and will also be recorded. The data of adverse events will be extracted from the original documents, reviewed by the responsible researcher and inserted in the study database. For the purposes of this study, the following definitions will be considered:

• Adverse event: any undesirable sign, symptom or clinical condition that occurs after the start of study therapy, even if it is not considered to be related to study therapy.

• Serious adverse event: any medical event that results in: death, adverse life-threatening surgical experience, hospitalization or prolonged hospitalization, persistent or significant disability/disability, or congenital anomaly. Important medical events that do not result in death, are life-threatening or require hospitalization can be considered as serious when, based on the appropriate clinical opinion, put the patient at risk or require medical or surgical interventions to avoid one of the results indicated in this definition.

4.10.2. Efficiency:

Endocervical canal stenosis (early and late) will be assessed through clinical information and physical examination at each visit to the hospital after surgery. The participant will be asked about menstrual flow and dysmenorrhea during anamnesis, which would indirectly show whether the endocervical canal is patent or not. In the physical examination, after viewing the cervix through specular examination, the doctor will check the permeability of the canal by introducing the hysterometer through the cervical canal. The difficulty of introducing the 2.5mm hysterometer through the channel will be noted according to the following scale:

The possible passage of the hysterometer?

( ) No difficulty

( ) Little / Moderate difficulty, however possible passage of the hysterometer

( ) Could not pass

4.10.3. Quality of life:

Quality of life questionnaires are used in clinical trials and include important outcomes such as postoperative pain and related symptoms. For these assessments, in the present study, the following instruments will be used:

a) Functional Assessment of Cancer Therapy - Cervix Cancer (FACT-Cx), version 4.0, in Portuguese (Annex 1).

b) Universal Pain Assessment Tools: comprises the Visual Analog Pain Scale (VAS), the Numeric Visual Pain Scale (EVN) and the Faces Pain Scale (EF) (Annex 2).

4.11. Study interruption rules:

Below are the study interruption criteria:

a) Registration of a single grade 5 adverse event at any stage of the study;

b) Record of two grade 3 or 4 adverse events in the first stage of the study;

c) Record of two grade 3 or 4 adverse events in the second stage of the study;

d) the benefit of using the device under interim analysis;

e) Identification of considerable risk to participants not provided in this research protocol.

In any of the aforementioned situations, the researcher, before interrupting the study, will communicate the Research Ethics Committee (CEP) of the Barretos Cancer Hospital and await the substantiated opinion, unless the risk to the participants is imminent. In the latter situation, the researcher will interrupt the study to safeguard the research participants and will communicate the CEP as soon as possible.

4.12. Study visits and data collection:

TABLE 1 summarizes the activities carried out in the study according to the time of the visits.

4.12.1. Baseline visit:

At the time of the initial visit, preoperative tests (blood count, serum biochemistry, coagulogram, chest X-ray, electrocardiogram and pregnancy test) and pre-anesthetic evaluation of women in whom CAF is indicated will be checked. This preoperative assessment is done on a routine basis and does not include specific aspects of the study. Women who are eligible for CAF and meet the study's eligibility criteria will be invited to participate in the survey. Those who agree will sign the Informed Consent Form at the initial visit.

During this visit, demographic and clinical information will be collected through interviews and consultation of medical records (Annexes 3). At this time, the quality of life questionnaires will also be applied.

4.12.2. Operative and postoperative visits:

On the day of surgery, a form will be filled out with the data pertinent to the surgical procedure. (Annex 4).

Postoperative visits will take place 15 days (1-PO) and 30 days (2-PO) after surgery. (Annexes 5 and 6). The acceptable windows of opportunity for visits will be ± 3 days. During these visits, the participants will undergo a medical consultation, when anamnesis and gynecological physical examination will be carried out. They will also answer the quality of life questionnaires. Any adverse event will be recorded on a specific form each post-operative visit in accordance with the Common Terminology Criteria for Adverse Events v3.0 (CTCAE V3). (Annex 7).

Participants who have the DUDA device will have it removed during the 2-PO visit during the gynecological examination.

4.12.3. Follow-up visits:

Follow-up visits will take place 3, 6, 12 and 18 months after surgery (respectively FUP-1, FUP-2, FUP-3 and FUP-4) according to our flow chart described below. The acceptable windows of opportunity for visits will be ± 10 days. During these visits, participants will undergo a medical consultation, when anamnesis and gynecological physical examination will be performed, which will include the collection of material for cervical cytology, hysterometry to assess the permeability of the endocervical canal and colposcopy to visualize the squamocolumnar junction. The degree of permeability of the channel will be recorded in a specific form. During follow-up visits, quality of life questionnaires will also be applied and adverse events will be recorded and follow-up information will be collected (Annex 8-11).

4.12.4. Surgery information:

The researcher will record the surgical times for carrying out the CAF and for implanting the device. Such records will be performed separately following the datasheet at the time of surgery.

TABLE 1 - Summary of study activities according to the moment.

|  | ***Baseline*** | **Surgery** | **1- FUP** | **2-FUP** | **FUP-1** | **FUP-2** | **FUP-3** | **FUP-4** |
| --- | --- | --- | --- | --- | --- | --- | --- | --- |
| **Moment** | - | Day 0 | 15 days | 30 days | 3 months | 6 months | 12 months | 18 months |
| **Time of delay** | - | - | ± 3 days | ± 3 days | ± 10 days | ± 10 days | ± 10 days | ± 10 days |
| **Elegebility** | X |  |  |  |  |  |  |  |
| **Consent Information** | X |  |  |  |  |  |  |  |
| **Randomization** | X |  |  |  |  |  |  |  |
| **Surgery (LEEP)** |  | X |  |  |  |  |  |  |
| **Device** |  | X |  |  |  |  |  |  |
| **Medical History** |  |  | X | X | X | X | X | X |
| **Gynecologic exam** |  |  | X | X | X | X | X | X |
| **Time of remove device** |  |  |  | X |  |  |  |  |
| **Pap smear** |  |  |  |  | X | X | X | X |
| **Hysterometry** |  |  |  |  | X | X | X | X |
| **Colposcopy** |  |  |  |  | X | X | X | X |
| **QOL** | X | X | X | X | X | X | X | X |
| **Adverse events** |  |  | X | X | X | X | X | X |

FICT = Informed Consent Form, CAF = High frequency surgery, QOL = Quality of life, EA = Adverse event

NOTE: The safety analysis of the device will be performed until the 30th postoperative day.

5. SAMPLE CALCULATION

5.1. First step:

For the safety analysis of the device, 25 women will be included, as advised by the National Ethics and Research Commission (CONEP).

5.2. Second stage:

The sample calculation assumed the premise of a cervical canal stenosis rate of approximately 15% to 20% after the CAF described in the literature and reduced to 6% with the use of an endocervical device according to the uncontrolled trial by Luesley et al (11) Considering this difference in stenosis rates (15-20% vs. 6%), an alpha error of 5%, a beta error of 20% and a balanced allocation 1: 1, the sample estimate ranges from 104 to 203 in each group. Therefore, this stage of the study will recruit 120 women in each arm, totaling 240 participants.

It is estimated that recruitment will take place between 1 to 3 weeks in the first stage and between

25 to 30 weeks in the second stage, since the potential for recruitment is 06 to 10 surgeries per week.

6. STATISTICAL ANALYSIS

The casuistry will be characterized by means of descriptive statistics. Quantitative variables will be described by means and standard deviation or median and 25-75 percentiles, depending on their distribution. Qualitative variables will be described using absolute and relative frequencies.

The rates of stenosis of the cervical canal will be compared between the groups at different times of observation from the moment 2-PO using the chi-square test or Fisher's exact test, depending on the values ​​observed in the contingency tables. Adverse events are characterized by means of absolute and relative frequencies, and inferences are made using a 95% confidence interval. The calculation of the scores of the domains of the FACT-Cx questionnaire will follow the guidelines of the manual of the organization FACIT (Functional Assessment of Chronic Illness Therapy). The scores will be compared between the groups at each moment of observation using the t-test or Mann-Whitney test, depending on the data distribution. In all statistical calculations, the level of significance considered will be 5%.

An interim analysis is foreseen in the transition from the first to the second stage of the study and when the casuistry of the second stage reaches 25%, 50% and 75% of the recruitment estimate.

7. INDEPENDENT DATA MONITORING COMMITTEE

The study will have an independent committee made up of statisticians, researchers with experience in clinical trials and doctors with expertise in the area of ​​interest. This committee aims to ensure safety for participants throughout the study. Based on data from the collection forms and interim analyses on the effectiveness of the device and safety, the committee will make a recommendation to continue or interrupt the study.

The transition from the first to the second stage of this study is particularly critical and will depend on the recommendation of continuity of this committee and the analysis of a partial (safety) report by the CEP/CONEP System.

8. ETHICAL ASPECTS

8.1. Special thematic area:

The present study fits into the special thematic areas “therapeutic equipment and devices, new or not registered in the country” and “new invasive therapeutic procedures”, which are provided for in CNS Resolution 466/12, respectively in items IX.4.3 and IX .4.4. After analysis and approval by the Research Ethics Committee of the Barretos Cancer Hospital, the protocol will be forwarded to the National Research Ethics Commission (CONEP).

This research does not involve other special thematic areas.

8.2. Consent Form:

Applicants eligible for the study will be invited to participate in the research, giving them the necessary explanations and the time necessary to clarify doubts for autonomous decision making. There will be two consent forms, one for the first stage of the study (without randomization) and another for the second stage (with randomization).

8.3. The weighting of risks and benefits:

Women who participate in the study and who use the DUDA device may, in theory, have reduced the chance of endocervical canal stenosis after CAF. However, there is still no certainty about this benefit. As in any other clinical trial that evaluates new devices, there is the possibility of the occurrence of adverse events, such as genital bleeding and uterine infection, and others that are not predicted. A previously published uncontrolled clinical trial with 33 women who used an endocervical device after conization did not report any complications related to bleeding or infection. Based on this essay, it is believed that research participants will not be exposed to excessive risks.

8.4. Biological material bank:

This study will not constitute a biobank or biorepository.

8.5. Infrastructure and human resources:

The Barretos Cancer Hospital has the appropriate infrastructure to perform the surgical procedures and to provide the necessary assistance to the participant in case of damages resulting from the research. The responsible researcher and the other researchers have experience in research in the field of oncological gynecology.

8.6. Data confidentiality:

The collection forms, as well as the database, will not contain information that can identify the research participant, preserving their privacy. Participants will be identified by codes and the master list with their identification will be held by the responsible researcher in a safe place.

8.7. Guaranteed access to the search result

As each participant will be accompanied on an outpatient basis (periodic visits) at the Barretos Cancer Hospital, not only because of the research but also because of their underlying disease (cervical intraepithelial neoplasia), at each visit the study doctor will perform a gynecological physical examination and speak to the participant about the existence or not of cervical stenosis. Thus, everyone will know, during the outpatient follow-up, if postoperative stenosis of the cervical canal occurred.

9. DETAILED BUDGET

The detailed budget can be found in TABLE 2.

TABLE 2 - Detailed study budget.

| FEATURE | DISCRIMINATION | **R$** |
| --- | --- | --- |
|  |  |  |
| Human resources Researcher | Salary | R$ 0,00 |
|  |  |  |
|  |  |  |
| Material resources | DUDA´s device – 170 units (*) | R$ 850,00 |
|  |  |  |
|  | Polypropylene surgical thread 2-0 - 170 units R $ 672.00 | R$ 672,00 |
|  | (14 boxes with 24 wires in each box) |  |
|  | Office supplies | R$ 500,00 |
|  |  |  |
|  | TOTAL | R$ 2022,00 |

(*) As it is an experimental device, the DUDA device has no price for commercialization. The figure presented represents the costs involved in making the 170 units

10. SCHEDULE

The study schedule after approval by the Research Ethics Committee is shown in Chart 3.

TABLE 3 - Study execution schedule.

| Study stage | Months elapsed after approval by the Research Ethics Committee | | | | | | | |
| --- | --- | --- | --- | --- | --- | --- | --- | --- |
|  | **1 – 3** | **4 – 6** | **7 – 9** | **10 – 12** | **13 – 15** | **16 – 18** | **19 – 21** | **22 - 24** |
| Inclusion (*1) | x | x |  |  |  |  |  |  |
| Follow up | x | x | x | x | x | x | x | x |
| Statistics Analysis (*2) | x | x |  |  |  |  | x | x |
| Preparation of the manuscript |  |  |  |  |  |  | x | x |
| Submission of the manuscript |  |  |  |  |  |  |  | x |

(* 1) The first stage of the study includes 25 participants for the safety analysis of the device (up to 30 days after surgery). The second stage will include 240 participants in each arm (randomized allocation: with and without the device). The recruitment estimate is 1 to 3 weeks in the first stage and 25 to 30 weeks in the second stage since the recruitment potential is 6 to 10 surgeries per week.

(* 2) An interim analysis is foreseen in the transition from the first to the second stage of the study and when the casuistry of the second stage reaches 25%, 50% and 75% of the recruitment estimate.

11. BIBLIOGRAPHY

1. Wright TC FA, Kurman RJ. Precancerous lesions of the cervix. In: Kurman RJ. In: Kurman RJ, editor Blaustein's pathology of the female genital tractNew York: Springer-Verlag. 2002.

2. Mitchell MF, Tortolero-Luna G, Wright T, Sarkar A, Richards-Kortum R, Hong WK, et al. Cervical human papillomavirus infection and intraepithelial neoplasia: a review. Journal of the National Cancer Institute Monographs. 1996(21):17-25.

3. Genest DR, Stein L, Cibas E, Sheets E, Zitz JC, Crum CP. A binary (Bethesda) system for classifying cervical cancer precursors: criteria, reproducibility, and viral correlates. Human pathology. 1993;24(7):730-6.

4. Wright TC, Jr., Cox JT, Massad LS, Carlson J, Twiggs LB, Wilkinson EJ, et al. 2001 Consensus guidelines for the management of women with cervical intraepithelial neoplasia. Journal of lower genital tract disease. 2003;7(3):154-67.

5. Monteiro AC, Russomano FB, Camargo MJ, Silva KS, Veiga FR, Oliveira RG. Cervical stenosis following electrosurgical conization. Sao Paulo medical journal = Revista paulista de medicina. 2008;126(4):209-14.

6. Baldauf JJ, Dreyfus M, Ritter J, Meyer P, Philippe E. Risk of cervical stenosis after large loop excision or laser conization. Obstetrics and gynecology. 1996;88(6):933-8.

7. Brun JL, Youbi A, Hocke C. [Complications, sequellae and outcome of cervical conizations: evaluation of three surgical technics]. Journal de gynecologie, obstetrique et biologie de la reproduction. 2002;31(6):558-64.

8. Ferris DG, Hainer BL, Pfenninger JL, Zuber TJ, DeWitt DE, Line RL. Electrosurgical loop excision of the cervical transformation zone: the experience of family physicians. The Journal of family practice. 1995;41(4):337-44.

9. Mathevet P, Chemali E, Roy M, Dargent D. Long-term outcome of a randomized study comparing three techniques of conization: cold knife, laser, and LEEP. European journal of obstetrics, gynecology, and reproductive biology. 2003;106(2):214-8.

10. Mathevet P, Dargent D, Roy M, Beau G. A randomized prospective study comparing three techniques of conization: cold knife, laser, and LEEP. Gynecologic oncology. 1994;54(2):175-9.

11. Luesley DM, Redman CW, Buxton EJ, Lawton FG, Williams DR. Prevention of post-cone biopsy cervical stenosis using a temporary cervical stent. British journal of obstetrics and gynaecology. 1990;97(4):334-7.

12. Nasu K, Narahara H. Management of severe cervical stenosis after conization by detention of nylon threads tied up to intrauterine contraceptive device. Archives of gynecology and obstetrics. 2010;281(5):887-9.

13. Grund D, Kohler C, Krauel H, Schneider A. A new approach to preserve fertility by using a coated nitinol stent in a patient with recurrent cervical stenosis. Fertility and sterility. 2007;87(5):1212 e13-6.
